# Assessment of the Winter Wheat Yield Gap for Smallholder Farmers in the Loess Plateau via Boundary Line Analysis

**DOI:** 10.3390/plants14213375

**Published:** 2025-11-04

**Authors:** Jing-Jing Han, Xian-Ping Xia, Hao Liu, Jia-Hui Wang, Ze-Wei Qi, Yue-Chao Wang, Wen Lin, Zhi-Qiang Gao, Shou-Tian Ma, Jian-Fu Xue

**Affiliations:** 1 Key Laboratory of Sustainable Dryland Agriculture of Shanxi Province, Shanxi Agricultural University, Jinzhong 030801, China; 2 Key Laboratory for Crop Water Requirement and Regulation, Institute of Farmland Irrigation, Chinese Academy of Agricultural Sciences (CAAS), Ministry of Agriculture and Rural Affairs of China, Xinxiang 453002, China

**Keywords:** boundary line analysis, yield gap, winter wheat, soil nutrients, Loess Plateau

## Abstract

Closing the yield gap in smallholder farming systems requires precise identification of key limiting factors. This study addresses this need by applying boundary line analysis (BLA) to diagnose primary soil constraints to winter wheat (*Triticum aestivum* L.) yield across 95 smallholder farms in the Loess Plateau of China. The BLA approach effectively delineates optimum nutrient ranges amidst inherent field variability, offering a novel methodological advantage for heterogeneous agricultural landscapes. The results showed that, regarding variability, the coefficients of variation for productive spike number and grain yield were considerably greater than those for kernels per spike and thousand-kernel weight. Soil available phosphorus showed the highest coefficient of variation (67.1%), 1.8–2.2 times greater than that of soil organic matter, alkali-hydrolyzed nitrogen, and available potassium. Boundary line models identified significant (*p* < 0.05) parabolic relationships, defining optimal ranges of 18.5–21.7 g kg^−1^ for soil organic matter, 10.4–49.0 mg kg^−1^ for alkali-hydrolyzed nitrogen, 40.5–61.6 mg kg^−1^ for available phosphorus, and 218.3–284.1 mg kg^−1^ for available potassium. Crucially, maintaining soil organic matter and available phosphorus within their respective optimal ranges was fundamental for maximizing yield. These findings provide a scientific basis for site-specific nutrient management and offer direct implications for designing targeted agricultural extension services and fertilization policies to enhance productivity in smallholder systems.

## 1. Introduction

Food security is a critical issue of worldwide concern for scientists, policymakers, and the public. Recent reports indicate that 713–757 million people suffered from hunger in 2023, affecting approximately one in eleven people globally and one in five in Africa [1]. Concurrently, with the global population projected to reach 8.5 billion by 2030 and 9.7 billion by 2050 [2], total food demand is expected to increase by 35% to 56% between 2010 and 2050 [3]. Boosting crop yield on existing farmland is therefore crucial for ensuring global food security.

This challenge is particularly pressing for China, which has a population exceeding 1.4 billion. Here, food security is intrinsically linked to social stability, economic development, and national security [4]. Enhancing the productivity of China’s existing cropland to bridge the gap between limited arable land and growing food demand is a fundamental strategy. However, significant yield gaps persist among regions and even among individual farms within the same region, largely due to variations in soil fertility and farm management practices [5,6]. Closing these yield gaps, especially at the smallholder level, is therefore a vital pathway for enhancing regional grain production capacity [7].

Soil nutrient status is widely recognized as a critical factor influencing the crop yield gap [5,8]. This is evident across diverse agricultural systems. For instance, a global meta-analysis by Oldfield et al. [6] demonstrated that increasing soil organic matter to region-specific targets could potentially increase average wheat yields by 23 ± 37% per hectare, thereby closing 60% of the global wheat yield gap. At the regional level, Khaliq et al. [9] identified nitrogen deficiency as the primary driver of the significant yield gap between farmer and potential yields for wheat in Pakistan. In practical agricultural production, soil nutrient levels can exhibit substantial variation even within the same region and soil type, contributing significantly to yield disparities among individual farms. Therefore, elucidating the relationship between soil nutrients and the crop yield gap at the farm level is crucial for developing targeted strategies to narrow this gap and enhance total grain production.

The yield gap among farmers stems largely from differences in key yield components—productive spike number, kernels per spike, and thousand-kernel weight—which are strongly influenced by spatially heterogeneous soil nutrients [10]. This relationship is well-documented across diverse wheat production systems. For instance, Cao et al. [11] identified the variation in spike number per unit area as the primary contributor to the yield gap among winter wheat farmers in the North China Plain. Similarly, Tanaka et al. [12], using structural equation modeling in Japan, demonstrated that spatially variable soil nutrients affect yield predominantly by modulating plant population and, consequently, spike number. Complementing these findings, Woźniak [13] reported that declining soil organic matter and total nitrogen content in Poland led to reductions in spike number, thousand-kernel weight, and kernels per spike, ultimately causing yield loss. Therefore, elucidating the role of farm-level soil nutrients in shaping the variation in yield components is essential for clarifying the fundamental causes of the yield gap among smallholders.

According to the Third National Land Survey, China has approximately 128 million hectares of arable land, with a per capita availability of only 0.09 hectares [14]. In this context of limited land resources, narrowing the on-farm yield gap by identifying its causes and implementing targeted improvements is crucial for enhancing total grain production and ensuring national food security. As one of China’s three major staple crops, wheat plays a pivotal role in the agricultural system of the Loess Plateau. In Shanxi Province, located in the eastern part of the plateau, wheat accounts for over 14.7% of the total crop sowing area and contributes about 16.7% to the province’s total grain output [15]. Therefore, understanding how soil nutrients influence the wheat yield gap and its components among smallholders in this region is essential for guiding efforts to increase regional wheat productivity and safeguard local food security. This study employs a survey-based statistical analysis in Wenxi County, a major winter wheat-producing county in Shanxi, to analyze the response of the yield gap and its components to soil nutrients. The objective is to identify key limiting soil factors and propose targeted soil fertility management practices to reduce yield disparities, thereby providing a scientific basis for regional food security.

## 2. Materials and Methods

### 2.1. Study Area

**Figure 1:**
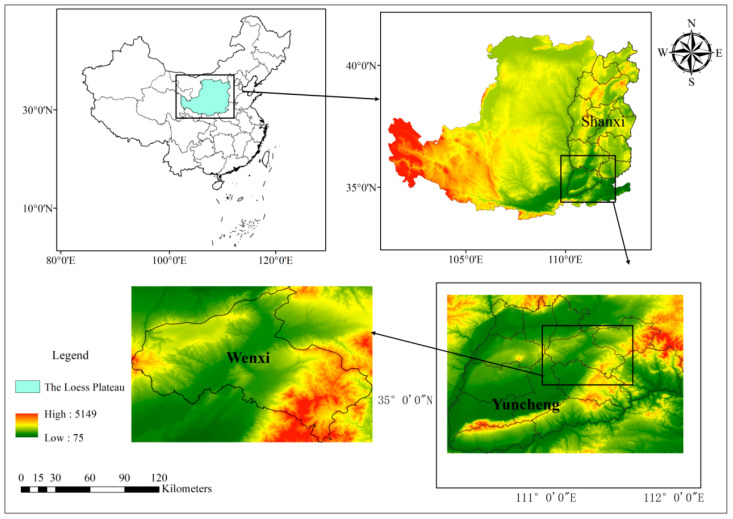
Geographical location of the study area.

This study was conducted in Wenxi County, located in Yuncheng City in the southern part of Shanxi Province, China (Figure 1). The county covers a total area of 1167.1 km^2^ and comprises 7 towns, 6 townships, and 342 villages. It is situated within the Huang-Huai-Hai wheat zone and experiences a warm temperate continental climate, characterized by a mean annual temperature of 12.5 °C and an average annual precipitation of approximately 506 mm, predominantly (60–70%) falling between July and September. The winter wheat growing season (October to June) is characterized by a cold winter and a typically dry spring, creating a common water-limited production environment on the Loess Plateau. The dominant soil texture across the surveyed area is loam, with pH values ranging from 7.5 to 8.5, indicating neutral to slightly alkaline conditions. According to the FAO classification system, the major soil type is Luvisol, which corresponds to Cinnamon soil (Haplic Luvisol) in the Chinese Soil Taxonomy.

As a major winter wheat-producing county in Shanxi, Wenxi had a sown area of 35,334 ha in 2023, ranking third in the province, with a total output of 167,068 t, ranking fourth [15]. The typical winter wheat cropping schedule in the county involves sowing from late September to mid-October and harvesting from early to mid-June the following year. The preceding crop for winter wheat in irrigated systems is most commonly summer maize. Conversely, non-irrigated cropland is maintained under a fallow state during the period from June to September.

### 2.2. Data Collection

A survey of winter wheat yield, along with plant and soil sampling, was conducted during the harvest season (1–8 June 2019). The investigation involved 102 randomly selected farm households across 18 villages in 7 townships of Wenxi County. An equal number of questionnaires (*n* = 102) were distributed, and corresponding wheat samples were collected. After screening the questionnaires and samples, those with incomplete information or that were lost were discarded, yielding 95 valid questionnaires and associated samples for final analysis.

For yield measurement, a representative 1 m^2^ area with uniform crop growth was selected in each farmer’s field. All spikes within this quadrat were collected to count the productive spike number, which was then converted to spikes per hectare (spikes ha^−1^). A random subset of 20 spikes was selected to determine the kernel number per spike. The total grain weight from the quadrat was determined after sun-drying and threshing. Subsequently, the thousand-kernel weight was measured, and the actual yield was calculated on the basis of a standard 13% grain moisture content.

Simultaneously with plant sampling, soil samples were collected from the 0–20 cm depth using a five-point sampling method within the same field. The samples were air-dried and sieved for subsequent soil nutrient analysis. Soil organic matter content was determined by the potassium dichromate oxidation method, the alkali-hydrolyzable nitrogen content by the alkali-diffusion method, the available phosphorus content by the 0.5 M sodium bicarbonate extraction method, and the available potassium content by the ammonium acetate extraction-flame photometry method [16].

Additionally, the crop management practices adopted by the farmers were investigated. A wide range of practices was recorded, including tillage, sowing density, fertilization, irrigation frequency, and the control of weeds, pests, and diseases. A detailed analysis of the effects of these management factors on yield is presented in a complementary study [17]. Cultivar information was also collected; however, the widespread use of self-saved seeds over consecutive years limited the reliability of varietal identification and precluded its systematic analysis.

### 2.3. Boundary Line Analysis

This study employed the boundary line analysis method to evaluate the effects of soil nutrient factors on winter wheat yield and its components under different smallholder farming conditions. The method was initially proposed by Webb in 1972 [18], which posits that the best-performing individuals within a population are located at the upper edge of the data distribution. A boundary line is derived by fitting these edge data points, on which the influence of extraneous factors is minimized, thus providing the optimal representation of the relationship between the two variables.

In this study, the contents of soil organic matter, alkali-hydrolyzable nitrogen, available phosphorus, and available potassium served as the independent variables, while winter wheat yield and its components (productive spike number, kernels per spike, and thousand-kernel weight) served as the dependent variables. The boundary line construction involved the following three steps:

(1) Data grouping: the dataset was partitioned into a yield group and a soil nutrient group. The yield group consisted of actual yield and its components, while the soil nutrient group consisted of the contents of soil organic matter, alkali-hydrolyzable nitrogen, available phosphorus, and available potassium.

(2) Boundary point identification: the data were categorized by soil nutrient concentrations into multiple levels. The boundary point for each level was determined by selecting the sample with the maximum yield value using the logical functions available in WPS Office.

(3) Boundary line fitting: an appropriate curve was fitted to the identified boundary points to establish the boundary line equation. Based on the resulting model, the optimal ranges of soil nutrient contents and yield components corresponding to maximum yield were subsequently determined.

### 2.4. Data Processing and Visualization

Data processing is performed using WPS Office (v12.1.0) software (Kingsoft Corporation, Beijing, China). Specifically, box plots were generated using Origin 2024 Pro (Learning Edition, OriginLab Corp., Northampton, MA, USA), while scatter plots and regression fittings were completed in WPS Office (v12.1.0) software. All statistical analyses were performed using SPSS 16.0 (IBM Inc., Armonk, NY, USA), with a significance threshold of *p* < 0.05.

## 3. Results

### 3.1. Variation in Winter Wheat Yield and Its Components

**Figure 2:**
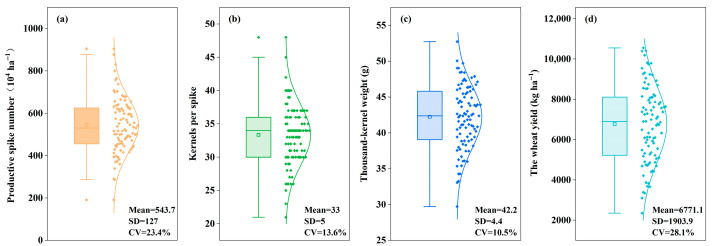
Winter wheat grain yield and its components across the surveyed farms in the study area. In parentheses, (**a**) indicates productive spike number, (**b**) indicates kernels per spike, (**c**) indicatesthousand-kernel weight, (**d**) indicates the wheat grain. Mean indicates the average value, SD indicates standard deviation; CV indicates the coefficient of variation.

The surveyed winter wheat crops showed substantial variation (Figure 2), with productive spike number ranging from 1.9 × 10^4^ to 9.1 × 10^4^ ha^−1^ (mean 5.4 × 10^4^ ha^−1^), kernels per spike from 21 to 48 (mean 33), thousand-kernel weight from 29.7 to 52.7 g (mean 42.2 g), and grain yield from 2353.5 to 10,558.1 kg ha^−1^ (mean 6808.6 kg ha^−1^). While the coefficients of variation (CV) for yield and its components ranged between 10.5% and 28.1%, among which productive spike number and grain yield exhibited the highest variability across different farms.

### 3.2. Soil Nutrient Distribution

**Figure 3:**
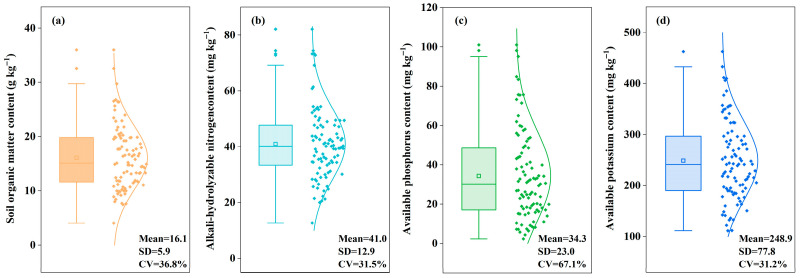
Soil nutrient variation in winter wheat fields across farms. In parentheses, (**a**) indicates soil organic matter content, (**b**) indicates alkali-hydrolyzable nitrogencontent, (**c**) indicates available phosphorus content, (**d**) indicates available potassium content. Mean indicates the average value, SD indicates standard deviation; CV indicates the coefficient of variation.

Soil nutrient analysis revealed considerable variation across the surveyed farmlands (Figure 3), with soil organic matter content ranging from 4.1 to 36.0 g kg^−1^ (mean 16.1 g kg^−1^), alkali-hydrolyzable nitrogen from 12.7 to 74.4 mg kg^−1^ (mean 40.6 mg kg^−1^), available phosphorus from 2.3 to 101.0 mg kg^−1^ (mean 34.3 mg kg^−1^), and available potassium from 111.3 to 462.7 mg kg^−1^ (mean 248.9 mg kg^−1^). The CV for these soil properties ranged from 30.0% to 67.1%. Notably, available phosphorus exhibited the highest variability (CV = 67.1%), which was 1.8–2.2 times greater than that of the other nutrients. In contrast, soil organic matter, alkali-hydrolyzable nitrogen, and available potassium showed relatively similar levels of variation.

### 3.3. Relationships Between Soil Nutrients and Yield Components

**Figure 4:**
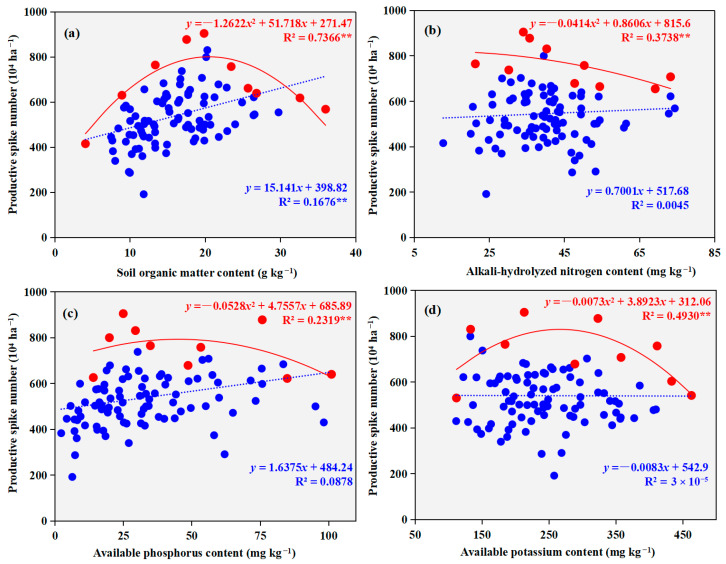
Regression relationships between winter wheat productive spike number and soil nutrient contents ((**a**) soil organic matter content; (**b**) alkali-hydrolyzable nitrogen content; (**c**) available phosphorus content; (**d**) available potassium content). Solid blue circles represent the survey data points; solid red diamonds indicate the boundary points identified through boundary line analysis. The blue dashed line shows the regression line fitted to all survey data, while the red solid line represents the boundary line fitted through the boundary points. *, *p* < 0.05, **, *p* < 0.01.

A significant positive linear correlation was observed between productive spike number and soil organic matter content (*p* < 0.01, Figure 4). In contrast, no significant relationships were detected between productive spike number and other soil nutrients (*p* > 0.05). Additionally, the relationship between productive spike number and soil nutrient levels was best described by a concave-downward quadratic polynomial (*p* < 0.01). Based on the boundary line analysis, the maximum productive spike numbers were achieved at soil organic matter, alkali-hydrolyzable nitrogen, available phosphorus, and available potassium concentrations of 20.5 g kg^−1^, 10.4 mg kg^−1^, 45.0 mg kg^−1^, and 266.6 mg kg^−1^, respectively. The corresponding peak productive spike numbers reached 8.01 × 10^6^ ha^−1^, 8.20 × 10^6^ ha^−1^, 7.93 × 10^6^ ha^−1^, and 8.31 × 10^6^ ha^−1^ under these optimal nutrient conditions.

**Figure 5:**
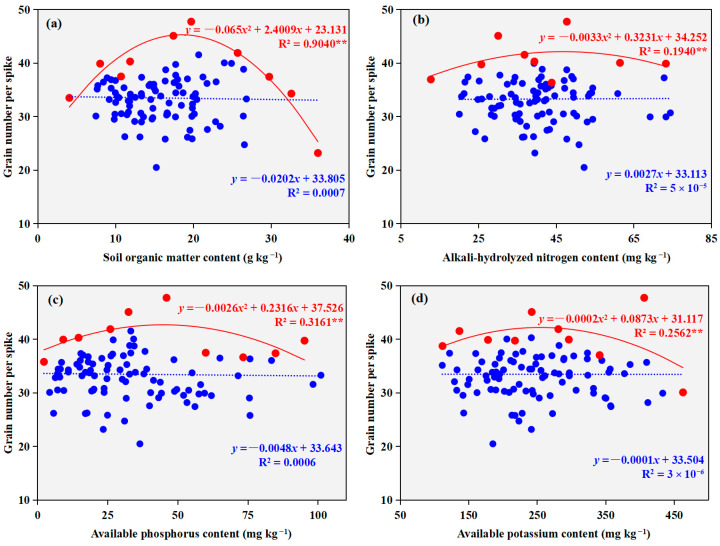
Regression relationships between winter wheat kernels per spike and soil nutrient contents ((**a**) soil organic matter content; (**b**) alkali-hydrolyzable nitrogen content; (**c**) available phosphorus content; (**d**) available potassium content). Solid blue circles represent the survey data points; solid red diamonds indicate the boundary points identified through boundary line analysis. The blue dashed line shows the regression line fitted to all survey data, while the red solid line represents the boundary line fitted through the boundary points. *, *p* < 0.05, **, *p* < 0.01.

No significant correlations were found between the surveyed soil properties and the grain number per spike of winter wheat (*p* > 0.05, Figure 5). In contrast, boundary line analysis demonstrated significant concave-downward quadratic relationships between these soil parameters and the grain number per spike *(p* < 0.01). The models indicated that the optimal grain number per spike was attained at soil organic matter, alkali-hydrolyzable nitrogen, available phosphorus, and available potassium concentrations of 18.5 g kg^−1^, 49.0 mg kg^−1^, 44.5 mg kg^−1^, and 218.3 mg kg^−1^, respectively. The corresponding maximum values for grain number per spike reached 45.3, 42.2, 42.7, and 40.6 grains under these optimal conditions.

**Figure 6:**
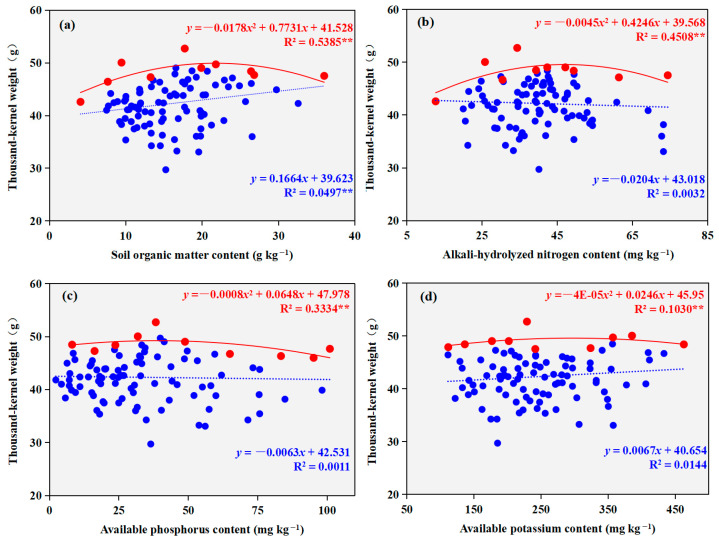
Regression relationships between winter wheat thousand-kernel weight and soil nutrient contents ((**a**) soil organic matter content; (**b**) alkali-hydrolyzable nitrogen content; (**c**) available phosphorus content; (**d**) available potassium content). Solid blue circles represent the survey data points; solid red diamonds indicate the boundary points identified through boundary line analysis. The blue dashed line shows the regression line fitted to all survey data, while the red solid line represents the boundary line fitted through the boundary points. *, *p* < 0.05, **, *p* < 0.01.

A significant positive linear correlation was identified between soil organic matter content and the thousand-kernel weight of winter wheat (*p* < 0.01, Figure 6), whereas no other soil properties showed significant correlations with it. Boundary line analysis revealed that the relationships of all tested soil properties with thousand-kernel weight were best fitted by significant concave-downward quadratic models (*p* < 0.01). The derived boundary lines indicated that the maximum thousand-kernel weight was achieved at concentrations of 21.7 g kg^−1^, 47.2 mg kg^−1^, 40.5 mg kg^−1^, and 246.0 mg kg^−1^ for soil organic matter, alkali-hydrolyzable nitrogen, available phosphorus, and available potassium, respectively. The corresponding peak thousand-kernel weights under these optimal nutrient conditions were 49.9 g, 49.6 g, 49.3 g, and 49.0 g.

**Figure 7:**
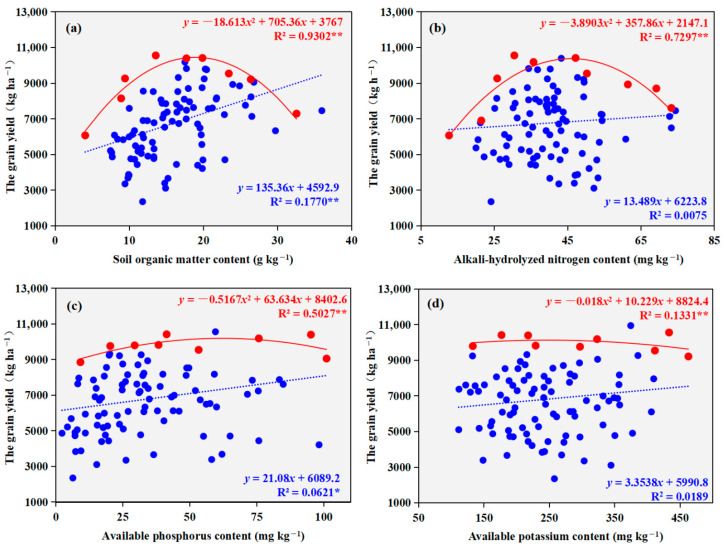
Regression relationships between winter wheat grain yield and soil nutrient contents ((**a**) soil organic matter content; (**b**) alkali-hydrolyzable nitrogen content; (**c**) available phosphorus content; (**d**) available potassium content). Solid blue circles represent the survey data points; solid red diamonds indicate the boundary points identified through boundary line analysis. The blue dashed line shows the regression line fitted to all survey data, while the red solid line represents the boundary line fitted through the boundary points. *, *p* < 0.05, **, *p* < 0.01.

Soil organic matter and available phosphorus content exhibited significant positive linear correlations with grain yield (*p* < 0.05, Figure 7), in contrast to other soil properties, which showed no significant associations. Boundary line analysis demonstrated that the relationships between all examined soil properties and yield were well-described by significant concave-downward quadratic models (*p* < 0.01). Based on these models, the optimum grain yield was achieved at soil organic matter, alkali-hydrolyzable nitrogen, available phosphorus, and available potassium concentrations of 18.9 g kg^−1^, 46.0 mg kg^−1^, 61.6 mg kg^−1^, and 284.1 mg kg^−1^, respectively. The corresponding maximum yield values under these optimal conditions reached 10,449.6, 10,376.8, 10,361.8, and 10,277.6 kg ha^−1^.

**Figure 8:**
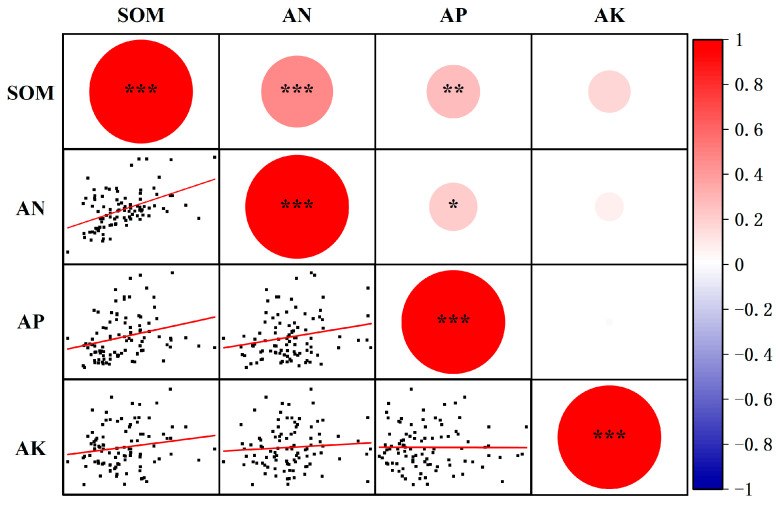
Correlation analysis among soil nutrient contents. SOM indicates soil organic matter content, AN indicates alkali-hydrolyzed nitrogen content, AP indicates available phosphorus content, and AK indicates available potassium content. *, *p* < 0.05, **, *p* < 0.01, ***, *p* < 0.001.

Significant positive correlations were observed between soil organic matter content and both alkali-hydrolyzable nitrogen (*p* < 0.001) and available phosphorus (*p* < 0.01), as well as between alkali-hydrolyzable nitrogen and available phosphorus (*p* < 0.05, Figure 8). In contrast, available potassium content showed no significant correlation with soil organic matter, alkali-hydrolyzable nitrogen, or available phosphorus.

## 4. Discussion

### 4.1. Variation in Yield Components and Underlying Causes

Analysis of winter wheat yield and its components in the surveyed region revealed that the coefficients of variation for grain number per spike and thousand-kernel weight were lower than those for productive spike number and grain yield. This pattern can be primarily attributed to the stronger genetic control exerted over grain number per spike and thousand-kernel weight [19]. In contrast, productive spike number and final yield are more susceptible to fluctuations in environmental conditions, such as climate and field management practices, leading to their higher variability [20]. Furthermore, the coefficients of variation for soil available phosphorus were notably higher than those of soil organic matter, alkali-hydrolyzed nitrogen, and available potassium. This disparity is likely associated with the inherently low mobility of phosphorus in soil, which often results in pronounced spatial heterogeneity, even within a confined geographical area [21].

### 4.2. Soil Organic Matter and Available Phosphorus as Key Yield Drivers

Regression analysis of the relationships between soil nutrients and winter wheat grain yield and its components revealed significant positive correlations of soil organic matter content with productive spike number, thousand-kernel weight, and grain yield. A significant positive correlation was also observed between soil available phosphorus and grain yield. The underlying mechanism may be that soil organic matter provides a stable and sustained supply of fundamental nutrients for winter wheat growth [22], which supports canopy establishment and the accumulation of dry matter in grains during the filling stage [23], thereby enhancing both productive spike number and thousand-kernel weight, and ultimately increasing grain yield. The significant influence of available phosphorus on grain yield can be attributed to two potential factors. First, its pronounced spatial variability within the study area (with a coefficient of variation of 67.1%, 1.8 to 2.2 times higher than that of other soil properties) may render phosphorus a limiting factor for grain yield enhancement in certain patches. Second, phosphorus plays a vital role in root system development [24]. Particularly under rain-fed conditions, an adequate phosphorus supply enhances the root capacity for soil water uptake [25], thereby supporting grain yield formation.

Furthermore, soil organic matter may indirectly improve phosphorus availability by complexing metal ions that would otherwise immobilize phosphorus [26]. Consequently, the impact of available phosphorus on winter wheat grain yield may be partially mediated by soil organic matter, although the specific mechanisms involved require further elucidation.

### 4.3. Non-Linear Nutrient Responses and Yield Inhibition at High Levels

Based on boundary line analysis of soil nutrients versus winter wheat yield and its components in farmer fields, the fitted boundary line equations all exhibited a concave-downward quadratic form, with statistically significant goodness-of-fit (*p* < 0.05). The results demonstrated that as soil nutrient levels increased, the grain yield and its components of the boundary data points initially rose and subsequently declined. This parabolic pattern may be attributed to a shift in the limiting factors governing yield formation as nutrient availability changes [27]. When nutrient concentrations fall below a specific threshold, nutrient supply acts as the primary constraint for grain yield [28]. However, once this threshold is exceeded, nutrients cease to be the main limiting factor, and their further accumulation may even suppress grain yield formation [28,29], necessitating the inclusion of additional environmental or physiological factors for a comprehensive explanation.

The mechanisms potentially responsible for yield inhibition under high nutrient conditions are multifaceted. One contributing factor involves elevated soil nutrient levels, particularly soil organic matter, which can intensify competition between vegetative and reproductive growth. This often results in excessive canopy density [30], delayed spike development, and compromised grain filling [31]. Beyond plant-internal competition, high nutrient availability can also intensify the competition for nutrients between soil microorganisms and the crop [32], potentially inducing transient shortages of available soil nutrients during critical phenological stages [33]. Furthermore, abundant soil nutrients, especially increased available phosphorus, can stimulate root proliferation [34]. However, excessive root development may lead to root redundancy [35], triggering competition for photosynthetic assimilates between above-ground and below-ground organs and consequently impairing yield [36].

### 4.4. Optimal Nutrient Ranges and Differential Responses of Yield Components

Based on further analysis of boundary line trends between soil nutrients and winter wheat yield components in farmer fields, it was found that when soil organic matter, alkali-hydrolyzed nitrogen, available phosphorus, and available potassium concentrations fell within the ranges of 18.5–21.7 g kg^−1^, 10.4–49.0 mg kg^−1^, 40.5–61.6 mg kg^−1^, and 218.3–284.1 mg kg^−1^, respectively, the corresponding roductive spike number, grains per spike, thousand-kernel weight, and grain yield reached high levels of 793.0–830.9 × 10^4^ ha^−1^, 40.6–45.3 grains, 49.0–49.9 g, and 10,277.6–10,449.6 kg ha^−1^. Notably, the optimal ranges for soil alkali-hydrolyzed nitrogen and available phosphorus were relatively wide. Specifically, the peak of the boundary line for alkali-hydrolyzed nitrogen corresponded to a concentration of 10.4 mg kg^−1^, which was only 21.2–22.6% of the optimal values for other yield components (49.0 mg kg^−1^ for grain number per spike and 47.2 mg kg^−1^ for thousand-kernel weight, respectively). This may be attributed to the fact that the formation of productive spikes primarily depends on canopy establishment during the early to middle growth stages of wheat [37], a period during which plants exhibit greater sensitivity to inorganic nitrogen fertilizer [38]. Additionally, the high nitrogen uptake efficiency by the developing canopy during this phase may lead to a relative decline in soil nitrogen supply capacity later in the season [39].

On the other hand, the optimal range of soil available phosphorus for grain yield was wider than that for individual yield components (with the peak concentration being 1.4–1.5 times higher than for other traits). This phenomenon may be linked to the pivotal role of phosphorus in grain filling and assimilate translocation. Phosphorus participates in key metabolic processes involving starch phosphorylase [40], phytin [41], and ATP [42], thereby promoting the transport of photosynthetic assimilates from leaves to developing grains [39,40], enhancing grain plumpness, and ultimately determining final yield [43].

The optimal nutrient ranges identified in this study, particularly for soil organic matter and available phosphorus, should be interpreted in the context of crop management practices to enhance their field applicability [44]. Improved irrigation scheduling may synergize with the water-retention capacity of soil organic matter, supporting canopy development and grain filling under rain-fed conditions. Meanwhile, higher planting densities could increase phosphorus demand during early growth stages, potentially narrowing the effective optimal available phosphorus range and necessitating adjusted phosphorus management strategies. Furthermore, the adoption of modern cultivars with enhanced nutrient-use efficiency may widen these operational ranges, offering greater adaptability under heterogeneous smallholder conditions. These interactions highlight the importance of developing integrated management strategies that combine optimized nutrient levels with improved agronomic practices to sustainably reduce yield gaps in smallholder systems.

### 4.5. Study Limitations and Future Research Perspectives

Based on field survey data from 95 smallholder farmers in Wenxi County, Shanxi Province, China, this study investigated the impact of soil nutrients on the winter wheat yield gap. While our boundary line analysis provides robust, data-derived optimal nutrient ranges, the interpretability of these findings is subject to certain methodological constraints. Given the limited variability in wheat management practices and socio-economic conditions across the studied county-scale region, this study did not explicitly address the effects of specific agronomic measures (e.g., seeding rate, irrigation, fertilization) or farmer socio-economic factors on the yield gap. Consequently, the identified optimal nutrient ranges should be viewed as foundational guidelines whose implementation may be moderated by unmeasured local management and economic contexts. Expanding the research to encompass a wider range of regional contexts is therefore warranted.

Field observations indicated that farmers generally placed limited emphasis on variety selection, with most being uncertain about the specific cultivars sown, leading to incomplete and potentially biased varietal data. Moreover, a large proportion of farmers continuously used self-saved seeds for one to three consecutive years, resulting in diminished seed purity and visibly reduced field uniformity and crop consistency. As a result, varietal differences among households were not included as an analytical factor in this study. This limitation, while constraining our ability to assess cultivar-specific effects, importantly highlights a widespread but often overlooked issue in smallholder systems—the significant yield penalty potentially associated with informal seed systems [45].

We quantitatively disentangle the complex parabolic relationships between multiple soil nutrients and yield components, providing a more integrated understanding of yield constraints in a real-world smallholder context. While this research provides initial insights into factors contributing to the yield gap at the farm level, and some findings are consistent with previous studies, the underlying mechanisms require further validation through well-designed field experiments. Future research would specifically investigate the interaction between the identified optimal nutrient ranges and key management factors such as seeding rates, irrigation regimes, and varietal selection to develop more integrated management recommendations.

In Shanxi Province, winter wheat cultivation is predominantly concentrated in the southern region, with Yuncheng city and Linfen city collectively accounting for 89.7% of the provincial planting area in 2023 [15]. As a major wheat-producing county within Yuncheng, Wenxi County is located at the intersection of Yuncheng and Linfen and features diverse topography, including mountainous areas and alluvial basins, enhancing its regional representativeness. Thus, the analysis of yield gaps in this county offers valuable, albeit partial, insights into yield variation patterns across southern Shanxi. Building on our findings, we recommend that future research priorities should include (1) controlled experiments to verify the causal mechanisms behind the observed nutrient-yield relationships and (2) multi-county comparative studies across Shanxi’s major wheat-producing regions to validate and refine the optimal nutrient thresholds established here. However, a comprehensive understanding of the mechanisms driving winter wheat yield gaps throughout the province will require systematically expanding research to include other major wheat-producing counties in Shanxi.

## 5. Conclusions

Based on a comprehensive farm survey and boundary line analysis in Wenxi County, Shanxi Province, this study elucidates the relationship between soil nutrients and the winter wheat yield gap in the Loess Plateau region. The main conclusions are as follows:

Soil organic matter and available phosphorus were identified as key limiting factors for the yield gap. Soil organic matter significantly influenced productive spike number, thousand-kernel weight, and grain yield, while the high spatial variability of available phosphorus rendered it a major yield-limiting factor in specific areas. In contrast, alkali-hydrolyzable nitrogen and available potassium showed less consistent or nonsignificant relationships with yield across the study area, suggesting their limitations as universal constraints under current management practices.

Boundary line analysis revealed significant parabolic relationships between soil nutrients and yield-related traits. Optimal yields and yield components were achieved within specific nutrient ranges: soil organic matter at 18.5–21.7 g kg^−1^, alkali-hydrolyzable nitrogen at 10.4–49.0 mg kg^−1^, available phosphorus at 40.5–61.6 mg kg^−1^, and available potassium at 218.3–284.1 mg kg^−1^.

These findings are applicable to other semi-arid regions of the Loess Plateau with similar soil and climatic conditions, where SOM and AP variability may similarly drive yield gaps. To optimize yield structure and reduce the yield gap, we recommend maintaining SOM and AP within their respective optimal intervals through targeted organic amendments and precision phosphorus fertilization. Furthermore, the quantified optimal nutrient ranges established in this study provide a scientific basis for developing region-specific, adaptive fertilization policies that can guide soil testing and formula-based fertilizer recommendations for smallholders, thereby enhancing both economic and environmental sustainability.

## Data Availability

Data are contained within the article.
